# Light oxygen isotopes in mantle-derived magmas reflect assimilation of sub-continental lithospheric mantle material

**DOI:** 10.1038/s41467-021-26668-z

**Published:** 2021-11-02

**Authors:** Jing-Yao Xu, Andrea Giuliani, Qiu-Li Li, Kai Lu, Joan Carles Melgarejo, William L. Griffin

**Affiliations:** 1 State Key Laboratory of Lithospheric Evolution, Institute of Geology and Geophysics, Chinese Academy of Sciences, 100029 Beijing, China; 2 Innovation Academy for Earth Science, Chinese Academy of Sciences, 100029 Beijing, China; 3 Institute of Geochemistry and Petrology, Department of Earth Sciences, ETH Zurich, 8092 Zurich, Switzerland; 4 College of Earth and Planetary Sciences, University of Chinese Academy of Sciences, 100049 Beijing, China; 5 Department of Mineralogy, Petrology and Applied Geology, Faculty of Earth Sciences, University of Barcelona, 08028 Barcelona, Spain; 6 ARC Centre of Excellence for Core to Crust Fluid Systems (CCFS) and GEMOC, Earth and Environmental Sciences, Macquarie University, Sydney, NSW 2109 Australia

**Keywords:** Petrology, Geochemistry

## Abstract

Oxygen isotope ratios in mantle-derived magmas that differ from typical mantle values are generally attributed to crustal contamination, deeply subducted crustal material in the mantle source or primordial heterogeneities. Here we provide an alternative view for the origin of light oxygen-isotope signatures in mantle-derived magmas using kimberlites, carbonate-rich magmas that assimilate mantle debris during ascent. Olivine grains in kimberlites are commonly zoned between a mantle-derived core and a magmatic rim, thus constraining the compositions of both mantle wall-rocks and melt phase. Secondary ion mass spectrometry (SIMS) analyses of olivine in worldwide kimberlites show a remarkable correlation between mean oxygen-isotope compositions of cores and rims from mantle-like ^18^O/^16^O to lower ‘crustal’ values. This observation indicates that kimberlites entraining low-^18^O/^16^O olivine xenocrysts are modified by assimilation of low-^18^O/^16^O sub-continental lithospheric mantle material. Interaction with geochemically-enriched domains of the sub-continental lithospheric mantle can therefore be an important source of apparently ‘crustal’ signatures in mantle-derived magmas.

## Abstract (web summary)

Isotopically light oxygen in mantle derived magmas is generally attributed to the occurrence of deeply subducted crustal material in the mantle. New analyses of olivine in kimberlites worldwide suggest that assimilation of lithospheric mantle during magma ascent is the source of isotopically light oxygen in these, and, potentially other continental magmas.

## Introduction

The oceanic lithosphere is continuously recycled into the convective mantle at subduction zones and this process contributes to the compositional heterogeneity of the deep Earth^1^. The compositions of mantle-derived magmas can help to elucidate the flux of crustal material transported in the deep Earth inasmuch effective tools are available to trace the occurrence of material processed at near-surface conditions in the mantle source of these magmas. Oxygen-isotope geochemistry has greatly contributed to the advancement of chemical geodynamics and tracing the occurrence of recycled crustal material in the mantle because substantial oxygen isotope fractionation can only occur at relatively low temperatures at or near the surface^2^. Conversely, mantle peridotites and fresh basalts from mid-ocean ridges show a very restricted range of δ^18^O values—e.g., 5.18 ± 0.28‰ for mantle olivine^3,4^. Thus, deviations of δ^18^O from typical mantle values are thought to provide robust evidence for recycled crustal material in the mantle source of igneous rocks, as previously shown for some continental flood basalts^5–8^ and ocean island basalts^9,10^. An exception is provided by crust-contaminated basalts, which can show δ^18^O values unlike typical mantle values due to assimilation of crustal material with light or, more commonly heavy, oxygen-isotope composition^11,12^. As an alternative, it has been suggested that low oxygen-isotope ratios in Archean komatiites may be inherited from primordial mantle heterogeneities^13^.

Some types of deep-mantle magmas erupted in intracontinental settings, such as flood basalts, kimberlites and ultramafic lamprophyres, traverse thick sub-continental lithospheric mantle (SCLM) roots to reach the upper crust. It is well established that during ascent these magmas widely interact with the lithospheric mantle^14–19^. For example, the composition of carbonate-rich kimberlite melts is considered to be partly controlled by the composition of SCLM wall rocks that are entrained and assimilated during ascent^20,21^. The lithospheric mantle beneath continental areas is known to host large variations in oxygen isotope compositions, which far exceed those recorded by typical depleted mantle peridotites. These include δ^18^O values between 2 and 12‰ in eclogites, metasomatised peridotites and more exotic metasomatic lithologies such as mica-amphibole-rutile-ilmenite-diopside (MARID) and phlogopite-ilmenite-clinopyroxene (PIC) rocks^22–26^. Although often overlooked, the heterogenous composition of the SCLM hampers a straightforward interpretation of oxygen-isotope signatures in deep mantle-derived magmas, which are unlike those of typical mantle rocks. A clear example is provided by oxygen-isotope variations in continental flood basalts, which have been attributed to the contribution of deeply subducted eclogites (or subduction-related metasomatism) either in the mantle source of these magmas or assimilated during magma ascent through the SCLM^5–8,15^. These contrasting interpretations have wide-ranging implications for understanding the cycling and storage of surface-derived volatiles delivered to the Earth’s mantle via subduction. Hence, a clearer understanding of the role of SCLM assimilation in modifying the oxygen isotope composition of deep-mantle magmas is required, which can also help elucidate whether or not the SCLM represents a significant reservoir of isotopically anomalous oxygen (i.e., compared to typical mantle values).

Kimberlites are potassic and ultrabasic igneous rocks^27,28^ rich in volatiles (H_2_O and CO_2_) and olivine and are considered to be the most deeply-derived magmas^29–33^. They are hybrid rocks comprising a mixture of mantle-derived and crust-derived xenoliths and xenocrysts, and crystals grown directly from the carrier kimberlite magma^28^. The location and composition of the mantle sources of kimberlites, including the contribution of deeply subducted recycled crustal material to the source of kimberlites, are unclear. High (HIMU-like) ^206^Pb/^204^Pb and unradiogenic Nd–Hf isotopic compositions in Mesozoic and Cenozoic kimberlites from southern Africa, Brazil, and Lac de Gras (western Canada) suggest the involvement of subducted oceanic crust in their sources^32,34–36^. However, in other regions such as Siberia and West Greenland, the Sr–Nd–Hf isotopic compositions of kimberlites are only marginally more depleted than those of the chondrite-based bulk silicate Earth^32,37–41^, which might not require recycled crustal material in their sources.

Previous oxygen-isotope analyses of olivine in Mesozoic and Cenozoic kimberlites from southern Africa, Lac de Gras, and Brazil did not exhibit any significant deviation from typical mantle values^42^, contradicting the evidence from radiogenic isotopes. Similarly, most oxygen-isotope analyses of garnet and zircon megacrysts, which share a common source with kimberlites based on isotopic and geochronological similarities^35,40,43^, exhibit typical mantle values in Mesozoic kimberlites from southern African, central-eastern North America and, in most cases, Brazil^44–46^. However, some of these Brazilian kimberlites, especially those from the Juina area, contain zircon megacrysts that have lower δ^18^O values than typical mantle values^46^. Similarly, low-δ^18^O zircon megacrysts were found in the Permian Jwaneng kimberlite (Botswana)^44^. Additionally, the metasomatic phases that cement mantle-derived polymict breccias (i.e., failed kimberlite intrusions at mantle depths^47^) from the Cretaceous Kimberley and Jagersfontein kimberlites (South Africa) exhibit oxygen-isotope compositions lower than those of similar phases (e.g., olivine and ilmenite) in typical mantle peridotites^48,49^. Based on this contrasting evidence, it is unclear whether oxygen-isotope variations in kimberlite-related products such as some zircon megacrysts and mantle-derived polymict breccias are related to isotopic variability in the kimberlite source or in the SCLM wall rocks.

Olivine is the most abundant mineral in kimberlites (~40–60% modal^27,28^) and is commonly zoned^50^. Olivine cores are considered to be mantle-derived xenocrysts, entrained from lithospheric mantle-wall rocks during ascent, based on their overlapping compositions with olivine in mantle peridotites, common resorption, and because they host inclusions of mantle-derived phases that are unstable in kimberlites (orthopyroxene, clinopyroxene, and garnet)^20,51–59^. Hence, olivine cores provide a powerful tool for examining the composition of the SCLM traversed by kimberlite magmas^21^. Olivine rims crystallize from kimberlite magmas during ascent, thus providing information about kimberlite composition at the early stages of their evolution^50,60^. Because of the different origins of these olivine zones, in situ analytical methods are required to investigate the origin of mantle-derived and magmatic components and their possible genetic relationships.

In this contribution these questions are addressed using a new oxygen-isotope dataset that includes in situ SIMS analyses of zoned olivine grains in nine Mesozoic kimberlites from southern Africa and central-eastern North America, two Mesoproterozoic Indian olivine-lamproites and two PIC-like xenoliths from the Cretaceous Damthsaa kimberlite in Botswana (Table 1 and Supplementary Fig. 1). PIC xenoliths were previously interpreted to represent the metasomatic products of kimberlite activity in the SCLM based on the overlap of Sr–Nd–Hf–Pb isotope compositions between PIC minerals and the kimberlite host^26,61^. We show that the average oxygen isotope composition of olivine cores and rims are directly correlated and extend to isotopic ratios lower than typical mantle values. These results suggest that assimilation of enriched lithospheric mantle material is a potential source of light oxygen isotope signatures in mantle-derived magmas from continental settings.

**Table 1:** List of samples studied in this work including geographic provenance, rock type, emplacement age and number of olivine analyses undertaken for each sample.

Sample	Locality	Rock type	Country, region	Craton	Age (Ma)	Error	Age source	N. EPMA analyses	N. SIMS analyses
10,060	Damtshaa BK9	VK	Botswana, Orapa	Zimbabwe, craton margin	94	10	^93^	24	46
10,049	Karowe AK6, South pipe	CK	Botswana, Orapa	Zimbabwe, craton margin	90–97		^93^	61	42
10,051	Karowe AK6, North pipe	CK	Botswana, Orapa	Zimbabwe, craton margin	90–97		^93^	23	11
10,059	Letlhakane DK1	CK	Botswana, Orapa	Zimbabwe, craton margin	94	6	^93^	30	12
9364	Dutoitspan	CK	South Africa, Kimberly	Kaapvaal, on craton	84–88		^93^	35	18
9630	Kaalvallei	CK	South Africa, Kaalvallei	Kaapvaal, on craton	100^a^	6	^93^	34	31
9607	P200	CK	Lesotho, north Lesotho	Kaapvaal, craton margin	(91–92^b^)		^93^	33	60
9352	Menominee Site 73	CK	USA, Michigan	Superior, craton margin	168		^62^	32	36
9353/ 9354	Notre Dâme du Nord	CK	Canada, Timiskaming	Superior, on craton	126		^94^	26	19
9360	Jackson Inlet	CK	Canada, Nunavut	Rae, on craton	111	8	This work^a^	20	18
8025	Wajrakarur P2	Lamproite	India, Wajrakarur	Eastern Dharwar, on craton	1124	5	^95^	9	9
8035	Wajrakarur P4	Lamproite	India, Wajrakarur	Eastern Dharwar, on craton	1023	40	^96^	11	13

VK volcaniclastic kimberlite, CK coherent (hypabyssal) kimberlite

^a^For Jackson Inlet, this is a new perovskite U/Pb age (Supplementary Fig. 2).

^b^For P200 the age is assumed based on that of the nearby Liqhobong pipe.

## Results and discussion

**Fig. 1:**
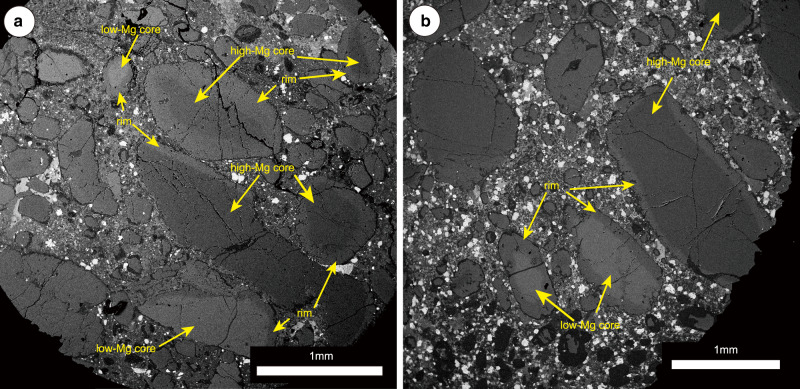
Back-scattered electron (BSE) SEM imaging of olivine in kimberlites. **a** Karowe AK6 South pipe (Botswana); **b** P200 (Lesotho). Note the strong core-rim zoning in kimberlitic olivine.

Back-scattered electron (BSE) images (see “Methods” section) show that in the studied samples olivine macrocrysts and microcrysts are commonly zoned between core and rim, with sharp variations in Mg# (Mg# = 100 × Mg/(Mg + Fe)). Two types of cores can be distinguished based on their BSE response, which reflects their composition: BSE-darker high-Mg cores and BSE-brighter low-Mg cores (Fig. 1 and Supplementary Fig. 3). Occasionally, internal zones are observed between core and rim in BSE images, a feature common to olivine in other kimberlites^50,58,60^. The internal zones and rims sometimes contain euhedral to subhedral inclusions of typical kimberlite groundmass minerals, such as spinel-group minerals and ilmenite, with compositions similar to those found in the kimberlite groundmass^62^.

### Major/minor element compositions

**Fig. 2:**
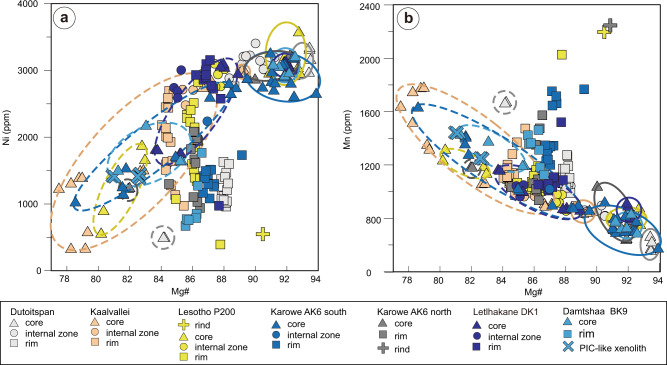
Mg#-Ni-Mn covariations diagrams for olivine grains. Mg# versus (**a**) Ni and (**b**) Mn in olivine from the examined southern African kimberlites. Solid lines mark out high-Mg olivine cores, and dashed lines the low-Mg olivine cores.

Major-element and minor-element concentrations were acquired by electron-probe microanalysis (EPMA; see “Methods” section). A Mg# value of 89 is proposed as the boundary between low-Mg and high-Mg olivine in this study because olivine in mantle peridotites entrained by kimberlites typically has Mg# > 89 (see refs. ^50,60^). The high-Mg cores (Mg# = 89.1–93.8, *n* = 80) have relatively high Ni (2460–3588 ppm) and Cr contents (average: 226 ppm, maximum: 958 ppm), and low Co, Zn, and Mn contents (Co = 116–152 ppm, Zn = 20–86 ppm, Mn = 563–1199 ppm). They show variable Ca contents (45–770 ppm), whereas Ti is relatively low (generally <200 ppm). In contrast, the low-Mg olivine cores (Mg# = 77.5–88.9, *n* = 49) show lower Ni (344–3113 ppm) and Cr contents (average: 127 ppm; maximum: 432 ppm), but higher Co, Zn, and Mn contents (Co = 123–184 ppm, Zn = 84–205 ppm, Mn = 862–1981 ppm) (Fig. 2 and Supplementary Fig. 4). They also show variable Ca and Ti contents (Ca =118–1193 ppm, Ti = 80–270 ppm). Olivine grains in the PIC-like xenoliths from Damtshaa have major/minor element compositions similar to those of low-Mg olivine cores.

The olivine rims show restricted Mg# ranges in each kimberlite (Fig. 2 and Supplementary Figs. 4 and 5). The Ni content of olivine rims is lower than that of high-Mg cores and within the range of low-Mg cores. In contrast, the Mn, Zn, and Co contents of olivine rims are higher than those of high-Mg cores and similar to or lower than those of low-Mg cores. The Ti contents of the rims are higher than those of both core types. The olivine rims show very variable Ca (104–4662 ppm) and, to a lesser extent, Ti contents (50–897 ppm). The internal zones between cores and rims have higher Mg# than the rims in most southern African kimberlites. In contrast, olivine internal zones feature lower Mg# than the rims in the Lethlakane and Menominee kimberlites.

### Oxygen-isotope compositions

**Fig. 3:**
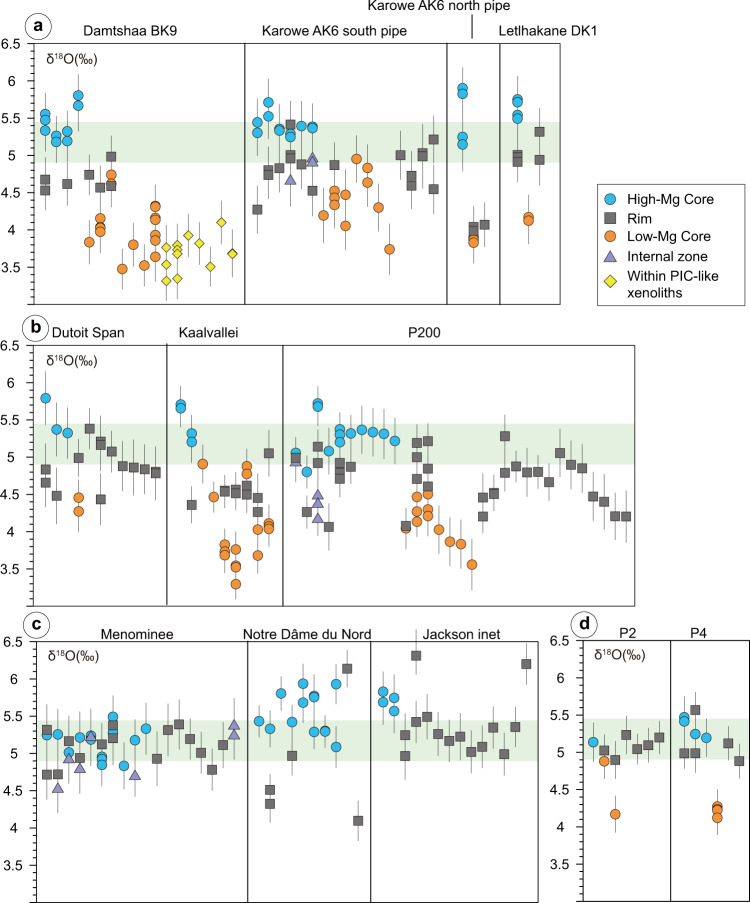
Oxygen isotope composition of olivine analyzed by SIMS in this study. Olivine in kimberlites from **a** Botswana, **b** South Africa and Lesotho, **c** North America, and **d** lamproites from India. Different olivine zones from the same grain are plotted on the same *x* coordinates. Green bands represent the mantle olivine value (5.18 ± 0.28‰; see ref. ^3^). Error bars indicate the 2*σ* of each analysis.

**Fig. 4:**
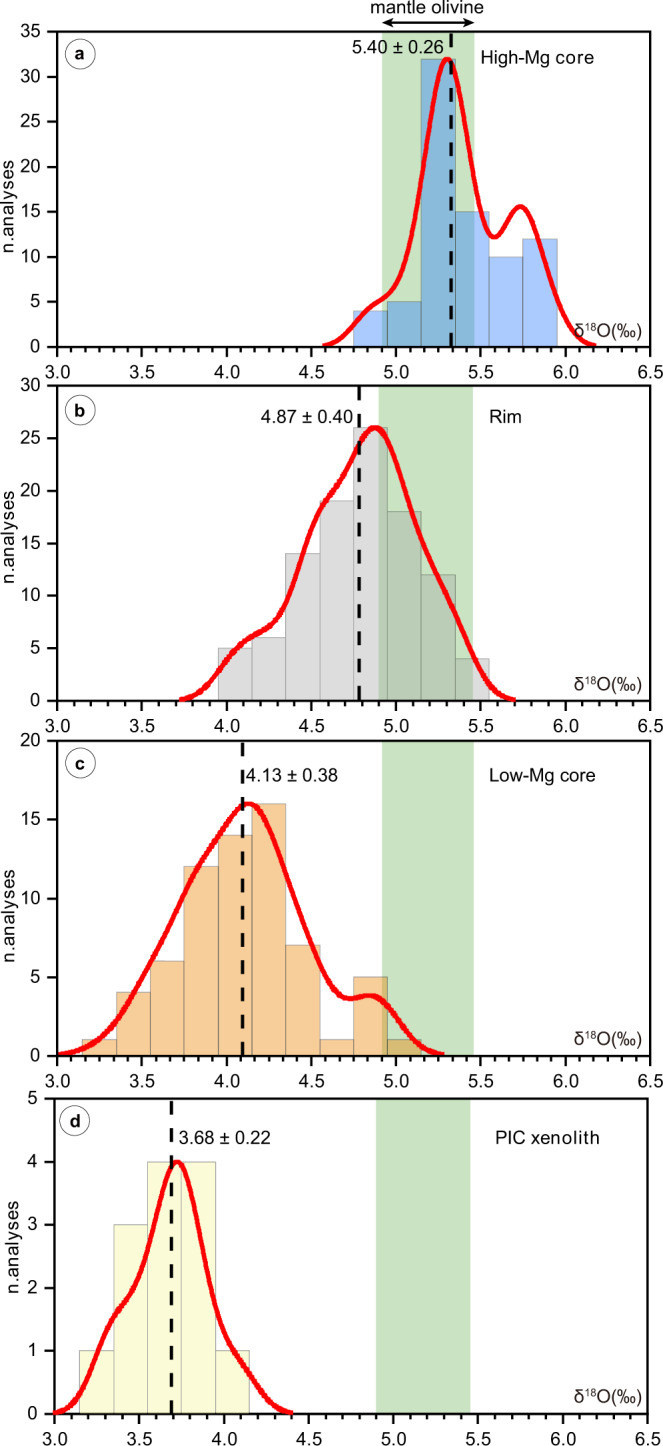
Probability distribution plots of oxygen isotope compositions in olivine from this study. **a** High-Mg olivine cores, **b** olivine rims, and **c** low-Mg olivine cores in the examined kimberlites and lamproites, and **d** olivine in PIC-like xenoliths. The dashed lines and associated numbers (±1 standard deviation) indicate average δ^18^O values for each probability distribution plot. Green bands represent the mantle olivine value (5.18 ± 0.28‰; see ref. ^3^).

We performed 314 in situ oxygen-isotope analyses of olivine by SIMS (see “Methods” section), including 84 high-Mg cores, 69 low-Mg cores, 134 rims, 14 internal zones, and 13 olivine in the PIC-like xenoliths. Most analyses show δ^18^O values close to or within the expected mantle olivine value of 5.18 ± 0.28‰ (see ref. ^3^; 2SD standard deviation of the mean; see Figs. 3 and 4 and Supplementary Data 1). The high-Mg cores (*n* = 84) show a prominent peak at ~5.4‰, consistent with typical mantle olivine, and a secondary peak at marginally higher oxygen isotope ratios (Fig. 4a). Low-Mg olivine cores have δ^18^O values consistently lower than those of high-Mg olivine cores in the same sample (Figs. 3 and 4). Low-Mg olivine cores from the southern Africa Cretaceous kimberlites show δ^18^O values ranging from 3.30 ± 0.19‰ (2*σ* = $\sqrt{(2{{{{{\rm{SE}}}}}})^{2} + (2{{{{{\rm{SD}}}}}})^{2}}$ where SE is the analytical standard error of each analyses and SD is the standard deviation of the analyses of the San Carlos olivine reference material in each session and represents the 95% confidence level) to 4.95 ± 0.29‰ (2*σ*), with an average of 4.14 ± 0.36‰ (one SD, *n* = 34) for Orapa (i.e., Damtshaa, Karowe, and Lethlakane), 4.02 ± 0.50‰ (one SD, *n* = 16) for Kaalvallei, and 4.11 ± 0.28‰ (one SD, *n* = 11) for Pipe 200. The low-Mg olivine cores from southern African kimberlites show a normal distribution, with an average value of 4.14 ± 0.38‰ (one SD, *n* = 63, Fig. 4), which is distinctly lower than the typical value for mantle olivine. Low-Mg olivine cores were not observed in the studied North American kimberlites. Low-Mg olivine cores (*n* = 6) from the two Indian olivine-lamproite samples also show low δ^18^O values from 4.12 ± 0.21‰ (2*σ*) to 4.27 ± 0.17‰ (2*σ*), except for one isotopically heavier result (4.88 ± 0.17‰, 2*σ*). Olivine in PIC-like xenoliths from Damtshaa has an average δ^18^O of 3.68 ± 0.22‰ (one SD, *n* = 13, Fig. 4).

The olivine rims from southern African kimberlites have δ^18^O values that are intermediate to those of high-Mg and low-Mg olivine cores. Despite the large variability, from 3.99 ± 0.33‰ (2*σ*) to 6.31 ± 0.30‰ (2*σ*), these analyses show a quasi-normal distribution, with an average value of 4.85 ± 0.41‰ (one SD, *n* = 123), which overlaps with the lower end of the mantle olivine δ^18^O range and shows a tail towards lighter isotopic values. Conversely, most of the olivine rims from the North American and Indian kimberlites have δ^18^O values similar to those of mantle olivine. Olivine grains from Notre Dame du Nord (North America) have a different distribution, with three of the five rims exhibiting δ^18^O values below the mantle olivine range.

**Fig. 5:**
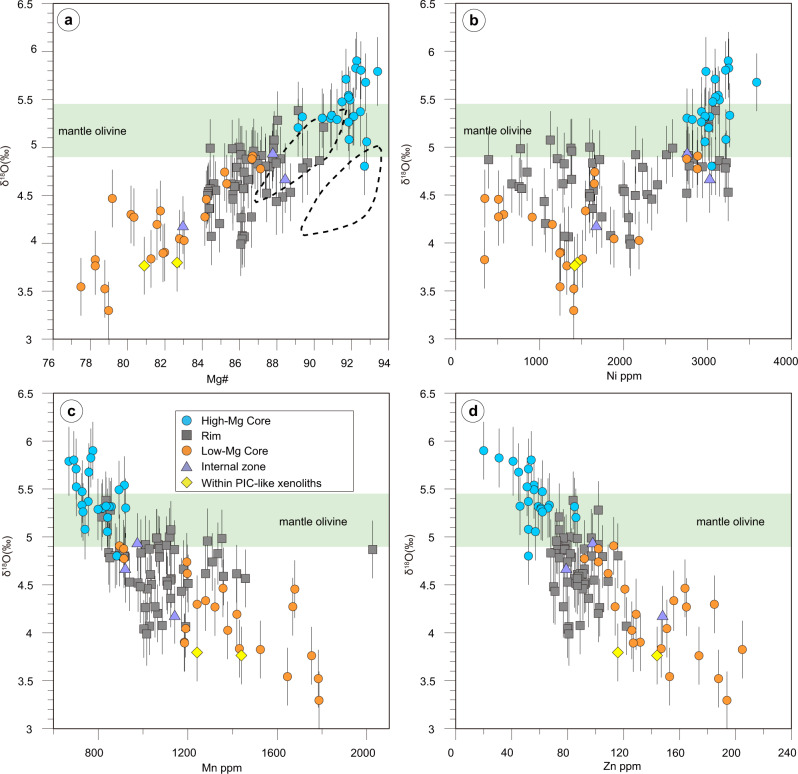
δ^18^O-Mg#-Ni-Mn-Zn covariation diagrams for olivine grains. Covariation diagrams of δ^18^O versus **a** Mg#, **b** Ni (ppm), **c** Mn (ppm), and **d** Zn (ppm) for olivine in the examined southern African kimberlites. Two dashed outlines show olivine compositions from two Kimberley polymict breccia xenoliths (i.e., failed kimberlite magmas at mantle depths)^48^. Green bands represent the mantle olivine value (5.18 ± 0.28‰; see ref. ^3^). Error bars indicate the 2*σ* of each analysis.

In the southern African kimberlites, olivine δ^18^O is positively correlated with #Mg and Ni content (Fig. 5a, b) and negatively correlated with Mn and Zn content when all the analysed zones are considered (Fig. 5c, d). Low-Mg olivine cores have lower δ^18^O and Ni contents but higher Mn and Zn contents than the high-Mg cores; the compositions of the olivine rims are intermediate between those of the two core types (Fig. 5). These correlations are also observed in samples from northern American kimberlites and Indian lamproites.

### Origin of high-Mg cores

Olivine zones in kimberlites have different origins, and the cores are considered to be representative of the mantle-wall rocks lining kimberlite magma conduits^60^. Major-element and trace-element compositions of most high-Mg olivine cores (Mg# > 89) match those of typical mantle olivine and plot within the field of olivine in coarse-grained granular peridotites (Supplementary Fig. 5, Mg# ~89–94; NiO ~0.30–0.45 wt%; CaO < 0.1 wt%; see refs. ^50,60,63,64^). Some analyses show Ca and Ti contents higher than olivine in typical cratonic peridotites, which probably suggest recent metasomatism before entrainment and overgrowth by kimberlite magmas^60,65^. The majority of high-Mg olivine cores analysed in this study show typical mantle δ^18^O values (5.18 ± 0.28‰; see ref. ^3^) within 2*σ* analytical uncertainties, consistent with an origin as mantle xenocrysts^50,52^. Some high-Mg cores (*n* = 15) in southern African and northern American samples exhibit slightly heavier oxygen-isotope compositions than typical mantle olivine (i.e., highest δ^18^O = 5.94‰ in Notre Dâme du Nord). These high-δ^18^O cores are restricted to very high-Mg# (>92) olivine with low Ti and Ca contents (Supplementary Fig. 6). These observations rule out a metasomatic origin for these anomalous oxygen isotope compositions and suggest that isotopically heavy oxygen can be locally associated with extreme mantle depletion.

### Fractionation of proto-kimberlite melts and the genesis of low-Mg olivine cores

The low-Mg olivine cores show a wide Mg# range from 89 to 77; the highest-Ni cores have high Mg# (close to 89) and low MnO contents (approximately 0.1 wt%) whereas the cores with lowest Ni contents show the highest Zn concentrations (Fig. 2). Their major-element and trace-element concentrations are consistent with those of olivine megacrysts in the Monastery and other South Africa kimberlites (Supplementary Fig. 6; see refs. ^50,66^). The low-Mg olivine cores have been suggested to be metasomatic products of proto-kimberlite (earlier, failed kimberlite) melts that interacted with peridotite wall rocks at mantle depths^50,57–59,67^ because of their compositional similarities with olivine megacrysts, as well as olivine in mantle polymict breccias and sheared peridotites^47,66,68,69^. Megacrysts are interpreted to be the product of fractional crystallization by failed kimberlite intrusions in the lower SCLM (1000–1400 °C, 4.5–5.5 GPa)^70,71^ not long before being entrained and transported to the upper crust by the host kimberlite. This interpretation is based on similar ages and radiogenic-isotope compositions of pyroxene, garnet and ilmenite megacrysts and the host kimberlite^35,43,72,73^.

The low δ^18^O values observed in low-Mg olivine cores are positively correlated with Mg# (Fig. 5a). Three hypotheses may explain the low δ^18^O and the decrease in δ^18^O with increasing Fe in these apparently megacrystic, low-Mg olivine cores: 1) derivation of the parental proto-kimberlite melt from a low-δ^18^O mantle; 2) isotopic fractionation during melt differentiation, potentially with attendant exsolution of CO_2_; and 3) interaction with low-δ^18^O wall rocks in the SCLM.

The cores of low-Mg olivine with Mg# close to 88–89 in this work represent some of the most primitive products of proto-kimberlite (“megacrystic”) melt crystallization (c.f. olivine megacrysts from Monastery^66^). These olivine cores have mantle-like δ^18^O (Fig. 5a). Similarly, garnet megacrysts from kimberlites worldwide show mantle-like δ^18^O (see ref. ^74^). For this reason, the hypothesis of a low-δ^18^O mantle source for the proto-kimberlite melt parental to low-Mg olivine from this study and megacrysts more generally can be ruled out.

Megacrysts are considered to have crystallized from fractionating magmas in the SCLM, starting with the crystallization of high-Mg olivine + orthopyroxene + clinopyroxene + garnet at ~1400 °C (ref. ^70^). The crystallization and fractionation of these minerals lead to a decrease in Mg# in the residual melt and olivine^73,75^. The effects of garnet and pyroxene fractionation on the oxygen-isotope composition of the melt that crystallized low-Mg olivine can be quantified using a Rayleigh distillation model and assuming an initial melt composition with mantle-like δ^18^O (5.4‰; see ref. ^76^), which fractionates the same proportions of olivine (Mg# = 90), orthopyroxene, clinopyroxene and garnet. The oxygen-isotope fractionation between olivine and melt is assumed to be −0.5‰ (as for Na-melilitite at 1400 °C; see ref. ^4^), and that between olivine and garnet, clinopyroxene, and orthopyroxene to be 0.20‰, 0.35‰, and 0.53‰, respectively (*T* = 1400 °C; see ref. ^4^). This model shows that fractionation leads to an increase in δ^18^O in the residual melt, and hence in olivine crystallized after early megacryst fractionation (Supplementary Fig. 8), which is the opposite of what observed in low-Mg olivine cores. Therefore, we can rule out the fractionation of megacrysts as a possible cause of oxygen-isotope variability in low-Mg olivine cores.

Assimilation of silicate minerals such as orthopyroxene and clinopyroxene in carbonate-bearing (proto-kimberlite) melts decreases the solubility of CO_2_ in the melt phase, triggering volatile exsolution^77^. Exsolution of a CO_2_-rich fluid can fractionate oxygen isotopes and decrease δ^18^O in the residual melt and crystallized olivine^78^. However, the model of Giuliani et al.^42^ shows that at temperatures similar to those considered here (1000–1400 °C), exsolution of 10 wt% CO_2_ cannot shift δ^18^O in crystallized olivine to less than 4.9‰ even assuming Rayleigh distillation. Considering extreme CO_2_ loss of 25 wt% (i.e., the highest CO_2_ contents estimated in primary kimberlite melts; see ref. ^79^ and references therein), δ^18^O in crystallized olivine cannot be more than ~1‰ lower than typical mantle values (i.e., ~4.2‰) if the melt was derived from a source with mantle-like oxygen isotope composition. These estimates are significantly heavier than the lowest δ^18^O values measured in low-Mg olivine cores (3.35‰), and preclude a dominant role of CO_2_ exsolution in the oxygen-isotope systematics of these olivine cores. In addition, at the high pressure of crystallization of these olivine cores (>4 GPa; see ref. ^70^) CO_2_ would be probably lost in a carbonate-rich melt rather than fluid/gas phase^80,81^ and oxygen-isotope fractionation between silicate-rich and carbonate-rich melts at high temperature is probably negligible.

Low-Mg olivine cores with higher Fe contents tend to have lower δ^18^O values (Fig. 5a). A correlation between radiogenic-isotope and major/trace element compositions has also been reported in clinopyroxene megacrysts from southern African kimberlites^82^. In that study the earliest crystallized megacrysts have Sr, Nd, and Hf isotope compositions similar to those of the entraining kimberlites, whereas those crystallized from more evolved melts at lower temperatures show more geochemically enriched signatures^82^, which are attributed to increasing interaction with strongly metasomatized SCLM. The oxygen-isotope systematics of low-Mg olivine cores could be ascribed to a similar process, in which the olivine cores with moderate Mg# (approximately 88–89) and mantle-like δ^18^O crystallized from pristine proto-kimberlite melts, whereas decreasing Mg# and δ^18^O (Fig. 5a) reflect increasing interaction with low-δ^18^O SCLM during melt differentiation. The question is then which SCLM material is involved in this assimilation process.

**Fig. 6:**
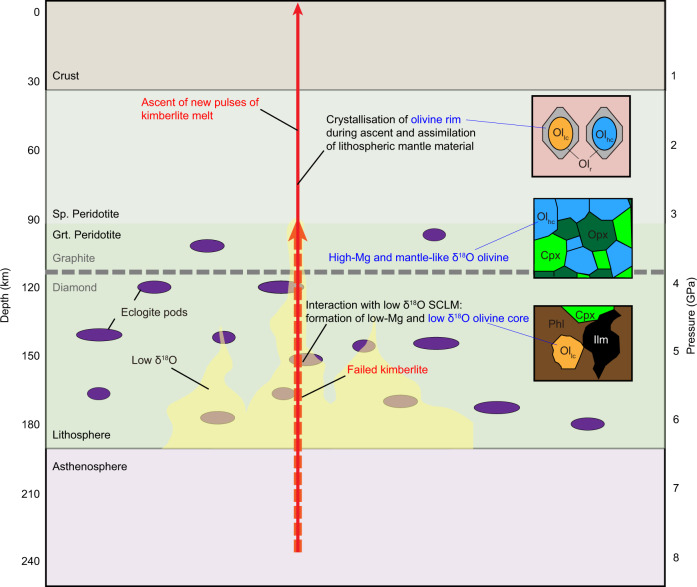
Schematic illustration showing the genesis of oxygen isotope variations in the cores and rims of kimberlitic olivine. High-Mg olivine cores with mantle-like δ^18^O represent xenocrysts derived from coarse-grained granular peridotites. Failed pulses of kimberlite magma may interact with low-δ^18^O metasomatic lithologies (± eclogites) in the sub-continental lithospheric mantle (SCLM) and generate low-Mg, low-δ^18^O olivine including olivine megacrysts and olivine in polymict breccias. Later pulses of kimberlite magma entrain wall-rock xenoliths and xenocrysts, including olivine, and crystallize olivine rims over cores of variable composition (both high-Mg and low-Mg). If the lithospheric mantle column traversed by kimberlite magmas is sufficiently enriched in metasomatised low-δ^18^O material, which is partially assimilated, the kimberlite melts crystallize low-Mg, low-δ^18^O olivine rims. Ol_lc_ low-Mg olivine core, Ol_hc_ high-Mg olivine core, Cpx clinopyroxene, Opx orthopyroxene, Phl phlogopite, Ilm ilmenite.

The only lithologies with oxygen-isotope compositions lower than typical mantle values that have been previously documented in the sub-cratonic lithosphere mantle are eclogites (as low as 2‰)^22,24,25^ and strongly metasomatised mantle rocks, such as phlogopite-bearing lherzolite and MARID (as low as 4.4‰ in clinopyroxene and 2.4‰ in ilmenite)^26^. However, most eclogites show oxygen isotopes heavier than typical mantle values (up to 12‰)^22,25^, especially those from Orapa, from where many low-δ^18^O olivine cores have been analyzed in this study. Moreover, it seems implausible that proto-kimberlite melts selectively interact with low-δ^18^O eclogites but not high-δ^18^O eclogites. For this reason, interaction with metasomatised mantle wall rocks is here preferred and is also more consistent with the radiogenic-isotope data of megacrysts in other southern African kimberlites^82^ (Fig. 6). The low δ^18^O values of these metasomatic lithologies were previously attributed to sourcing of the metasomatic agents in deeply subducted, hydrothermally altered oceanic lithosphere^83^ even though some contribution from low-δ^18^O mantle reservoirs inherited from early-Earth differentiation^13^ cannot be discounted.

Low δ^18^O values similar to those observed in low-Mg olivine cores from the southern African Cretaceous kimberlites also occur in olivine from PIC-like xenoliths from Damtshaa (Orapa; this study) and metasomatic minerals (olivine, phlogopite, and ilmenite) in mantle polymict breccias from the Kimberley and Jagersfontein kimberlites in southern Africa (δ^18^O as low as 3.8‰ in olivine)^48,49^. A positive correlation between δ^18^O, Mg# (Fig. 5a) and other trace elements in olivine was also observed in polymict breccias^48^. Therefore, the model of proto-kimberlite melt interaction with low-δ^18^O wall rocks in the SCLM that we have put forward to explain the origin of low δ^18^O in (megacrystic) low-Mg cores in this study may also explain the origin of low δ^18^O in PIC-like xenoliths and mantle polymict breccias, which have an origin similar to that of megacrysts^69^. In summary, this study illuminates an important role for lithospheric-mantle interaction in shaping the (isotopic) composition of mantle metasomatic products related to early (proto-)kimberlite activity including megacrysts, polymict breccias and PIC rocks. It is possible that this conclusion can be extended to mantle metasomatism elsewhere, a question which should be addressed by future studies.

### Origin of oxygen-isotope variations in kimberlites due to assimilation of sub-continental lithospheric mantle

Some of the olivine rims from the southern African kimberlites analyzed in this study have oxygen-isotope compositions slightly lower than typical mantle olivine values (26 of 89 samples analyzed have δ^18^O < 4.9‰, including a 2*σ* uncertainty, Fig. 4). δ^18^O values lower than typical mantle values are also observed in three olivine rims from Notre Dame du Nord (north America). Although contamination by hydrothermally-altered crust is a possible cause of low δ^18^O in oceanic basalts and their olivines^11,12^, olivine rims in kimberlites are believed to have crystallized before emplacement in the upper crust^50,60^ (Fig. 6). Therefore, low δ^18^O in the rims of kimberlitic olivine can result either from melt sources that carry isotopically-light oxygen of likely subducted crustal origin; or from the assimilation of low-δ^18^O material, including metasomatized mantle xenoliths (phlogopite-bearing lherzolite, MARID), low-Mg olivine cores, and perhaps other lithologies (e.g., eclogites) during kimberlite ascent. If the latter interpretation is correct, the oxygen-isotope composition of the olivine rims should be correlated with geochemical proxies of the local SCLM wall rocks which were entrained and assimilated.

**Fig. 7:**
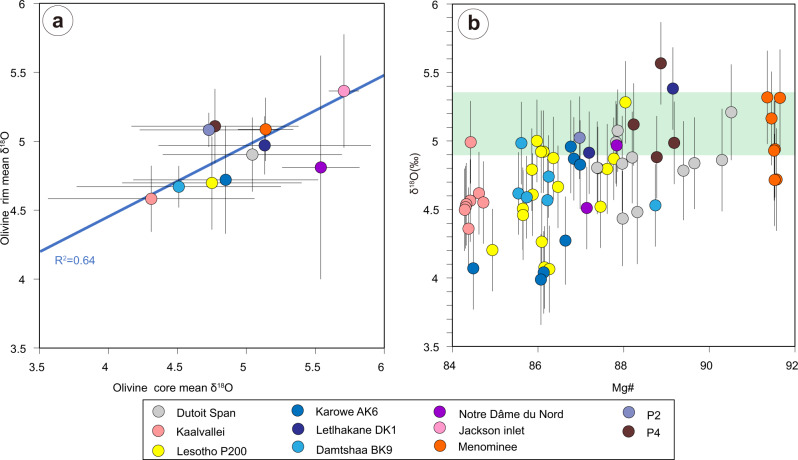
Relationships between the oxygen-isotope composition of olivine rims and cores, and the major-element composition of olivine rims. **a** Average olivine rim δ^18^O versus average olivine core δ^18^O in the kimberlites (and lamproites) from this study. Error bars are one SD of the mean. The blue line is the linear regression through the data points (excluding the Notre Dâme du Nord outlier). **b** Covariation diagram showing a broad direct correlation between δ^18^O and Mg# in olivine rims from the examined samples. Error bars indicate the 2*σ* of each analysis.

Resorption features on pyroxene, garnet, and olivine xenocrysts entrained by kimberlites worldwide^19,50,56,84^ provide clear evidence that kimberlite melts are modified by assimilation of entrained silicate minerals during ascent^21,51,54,77^. Furthermore, the mean values of Mg# in olivine rims are positively correlated with the mean Mg# values of olivine (xenocrystic) cores and negatively correlated with the abundance of low-Mg (xenocrystic) cores in kimberlites worldwide, which suggests that the Mg# composition of olivine rims in kimberlites is controlled by the composition of entrained (and assimilated) SCLM material^21^. When kimberlite magmas interact with Fe-rich SCLM material, including megacrysts and other metasomatised lithologies, olivine rims become enriched in Fe, Mn and Ti compared to kimberlites elsewhere^60^. In our study, olivine rims with δ^18^O values below the mantle range occur in kimberlites with large amounts of low-δ^18^O, low-Mg olivine cores of metasomatic origin (Fig. 3). Figure 7a shows a statistically robust direct correlation between the mean δ^18^O values of olivine rims and those of olivine cores in the examined kimberlites (*R*^2^ = 0.64; *n* = 10/11). This correlation provides firm evidence that the assimilation of low-δ^18^O SCLM material including low-δ^18^O, low-Mg “megacrystic” olivine generates the low δ^18^O values observed in olivine rims of some kimberlites. This process explains the occurrence of low-δ^18^O values in Fe-rich olivine rims from Orapa (Botswana), Kaalvallei (South Africa), and Pipe 200 (Lesotho), where olivine cores are dominated by Fe-rich compositions, and more broadly the correlation between δ^18^O and Mg# in olivine rims from the examined kimberlites (Fig. 7b). Conversely, kimberlite melts that have not extensively interacted with metasomatically-enriched low-δ^18^O lithospheric mantle have olivine rims with mantle-like oxygen isotope compositions.

In conclusion, this systematic examination of the major-element and minor-element and oxygen-isotope compositions of mantle-derived olivine cores and magmatic olivine rims in kimberlites shows that interaction with lithospheric mantle wall rocks is the primary source of oxygen-isotope variability in the examined kimberlites as well as in their precursor crystallization products at mantle depths (i.e., low-Mg olivine cores probably related to the megacryst suite). This conclusion could be extended to many products of mantle metasomatism in the ancient sub-continental lithospheric mantle (SCLM), a hypothesis which deserves further exploration. There is no conclusive evidence from the oxygen isotopes for the occurrence of recycled crustal material in the deep-mantle source of the examined kimberlites. We stress that the opposite conclusion would have been reached if the magmatic olivine rims had been considered in isolation, without addressing the oxygen-isotope composition of the lithospheric mantle that is traversed by these magmas. This study identifies the sub-continental lithospheric mantle as a reservoir of locally abundant isotopically-light oxygen which, as previously proposed for carbon stored in this mantle reservoir^85^, can be remobilised by ascending magmas. It further highlights that apparently “crustal” isotopic signatures in convective mantle-derived magmas such as kimberlites do not necessarily reflect recycling of crustal material in the magmatic source, i.e., one of the basic principles of chemical geodynamics, but can arise from interaction with metasomatic domains and/or eclogites in the SCLM. The examination of mantle-derived magmas, especially those emplaced above thick continental lithosphere, should therefore be paired with a deep understanding of the composition of the lithospheric mantle that these magmas traverse. Such an integrated approach provides the basis for a correct interpretation of the origin of “crustal” signatures in mantle-derived magmas, which has major implications for understanding the global cycling of volatiles elements between surface and deep Earth reservoirs.

## Methods

### Samples and petrography

We selected samples that contain abundant fresh olivine microcrysts and macrocrysts (Table 1 and Supplementary Fig. 1). The nine samples considered in this study include six Cretaceous kimberlites from the Kalahari Craton (Damtshaa, Karowe and Lethlakane in Botswana, Pipe 200 in Lesotho, and Dutoitspan and Kaalvallei in South Africa), one Jurassic and one Cretaceous kimberlite from the Superior Craton (Menominee in USA and Notre Dam du Nord in Canada), and one Cretaceous kimberlite from the Rae Craton (Jackson Inlet in Canada). Two Wajrakarur lamproites (P2 and P4) from the Eastern Dharwar Craton (India) were also analyzed for comparative purposes.

The studied samples are all coherent hypabyssal kimberlites, except for a volcaniclastic kimberlite from Damtshaa (also called Orapa BK-9; Botswana). All the hypabyssal samples exhibit an inequigranular texture wherein macrocrysts (>1 mm) and microcrysts are set in a fine-grained groundmass. Macrocrysts are generally anhedral and include olivine with occasional phlogopite, ilmenite, garnet, orthopyroxene, and clinopyroxene; whereas euhedral to subhedral microcrysts comprise olivine and phlogopite. The groundmass includes carbonates, serpentine, perovskite, spinel-group minerals, phlogopite, monticellite, and apatite. In the Damtshaa volcaniclastic kimberlite, only olivine microcrysts were selected for analysis.

Mantle xenoliths were occasionally present in the studied samples. Some phlogopite–ilmenite–clinopyroxene (PIC)^36,61,86^ mantle xenoliths have been observed in the Damtshaa kimberlite. We examined olivine grains in two of these PIC-like xenoliths, including a micro-xenolith (10 × 15 mm) consisting of olivine, clinopyroxene, phlogopite, and ilmenite, and a small xenolith (10 × 25 mm), in which clinopyroxene and orthopyroxene are also present. In both samples, phlogopite has a composition typical of PIC mica^86^.

### Electron-probe microanalyses

Petrographic studies and examination of olivine zoning were first performed on carbon-coated thin sections using a scanning electron microscope (SEM) equipped with a backscattered electron (BSE) detector. SEM-BSE imaging was performed using an E-SEM-BSE Quanta 200 FEI-XTE-325/D8395 instrument coupled to a Genesis energy dispersive spectrometer (EDS) at the Scientific and Technical Center of the University of Barcelona (CCiTUB).

Major and minor elements in olivine were analyzed using a CAMECA SXFive electron probe microanalyzer (EPMA) at the Institute of Geology and Geophysics, Chinese Academy of Sciences (IGGCAS). An acceleration voltage of 25 kV and beam currents of 900 and 40 nA were used for analyzing minor elements (Ni, Mn, Co, Zn, Ca, Ti, Al, Cr, Na, and P) and major elements (Si, Mg, and Fe), respectively, with a spot size of 5 μm. Diffracting crystals used for the analyses include: two TAP for Si, Mg, Na, and Al (Kα); a LIF for Fe, Cr, and Co (Kα); a LPET for Ca and Ti (Kα); and a LLIF for Mn, Ni, and Zn (Kα). Peak Counting times include 20 s on peak for Si, Mg, and Fe; 120 s for Na, Cr, Ca, Mn, Ni, and Zn; and 240 s for Co, Ti, and Al. The calibration standards used for analysis were natural halite for Na, natural rhodonite for Si, Ca, and Mn, synthetic periclase for Mg, natural specularite for Fe, synthetic Cr_2_O_3_ for Cr, synthetic rutile for Ti, synthetic corundum for Al, synthetic Ni metal for Ni, natural apatite for P, synthetic Co metal for Co and synthetic sphalerite for Zn. Instrumental drift was monitored by analysing San Carlos olivine twice every 30 analyses. Matrix correction and elemental quantification were undertaken using the phi-rho-Z matrix correction^87^. Oxygen was calculated by stoichiometry and included in the matrix correction. The high accuracy of the EPMA method for minor elements in olivine was previously confirmed by comparing the results of EPMA analyses with data from laser ablation inductively coupled plasma mass spectrometry (LA-ICP-MS)^88^. The following detection limits were based on a 3*σ* estimate of the measured background variance: 12 ppm for Ni, 14 ppm for Mn, 12 ppm for Co, 16 ppm for Zn, 6 ppm for Ca, 4 ppm for Ti, 10 ppm for Al, 20 ppm for Cr, 30 ppm for Na, 24 ppm for P, 240 ppm for Mg, 180 ppm for Si, and 200 ppm for Fe.

### SIMS oxygen isotope analyses

After the SEM and EPMA analyses, the carbon coating was removed. Selected portions of the thin sections were then drilled out and mounted in epoxy resin together with fragments of the oxygen isotope reference material San Carlos olivine. The San Carlos fragments are from one piece of mantle peridotite, and their δ^18^O value has been determined by laser fluorination in this work (δ^18^O = 5.20‰, *n* = 5, 2 SD = 0.07‰). The mounts were polished and coated with gold for in situ SIMS analysis. Oxygen isotope analyses of olivine were performed using a CAMECA IMS-1280 multi-collector ion probe at IGGCAS, using the procedure described by previous works^89,90^. The spot size was ~20 µm (10 µm beam diameter + 10 µm raster). An electron gun was used to compensate for sample charging during the analysis. Secondary ions were extracted at a −10 kV potential. Oxygen isotopes were measured in multi-collector mode with two off-axis Faraday cups, with each analysis consisting of 16 cycles with a counting time of 4 s. The reference material San Carlos olivine was analysed after every four unknown samples to monitor analytical precision and to calibrate instrumental mass fractionation. The two SD of δ^18^O values for San Carlos olivine measured by SIMS was 0.17–0.32‰ in all sessions. Previous studies have undertaken detailed work on SIMS matrix effects showing oxygen-isotope variations in olivine as a function of Fe molar fractions (e.g., ref. ^91^ and references therein) and demonstrated a parabolic correlation between instrumental fractionation of ^18^O/^16^O and Fe/(Fe + Mg). In olivine with Mg# of 100–75, instrumental fractionation was found to be too small to be resolvable. By comparing the results of laser fluorination and SIMS analyses of in-house olivine standards with a range of Mg# compositions (Supplementary Data 2), the present work confirms a negligible matrix effect (<0.1‰) in the oxygen isotope analysis of olivine with Mg# >80 using the CAMECA IMS 1280 ion probe at IGGCAS (Supplementary Fig. 9, and also see ref. ^92^). This Mg# interval covers the compositional range of olivine unknowns analysed in this study. In summary, instrumental mass fractionation is negligible for the oxygen-isotope ratios of olivine samples analysed in this work.

## Supplementary information

Supplementary Information

Supplementary Data 1

Supplementary Data 2

Supplementary Data 3

## Data Availability

The data analysed or generated in this study are included in this published article (and its supplementary Data). Source data supporting the plots are provided with this paper.
