# Supplementary material for: Light oxygen isotopes in mantle-derived magmas reflect assimilation of sub-continental lithospheric mantle material
Source: Nat Commun. 2021 Nov 2;12:6295. doi: 10.1038/s41467-021-26668-z (PMC8563987; doi:10.1038/s41467-021-26668-z)
Supplement: Supplementary file 1 — Supplementary Information [file 41467_2021_26668_MOESM1_ESM.pdf]

Supplementary Materials

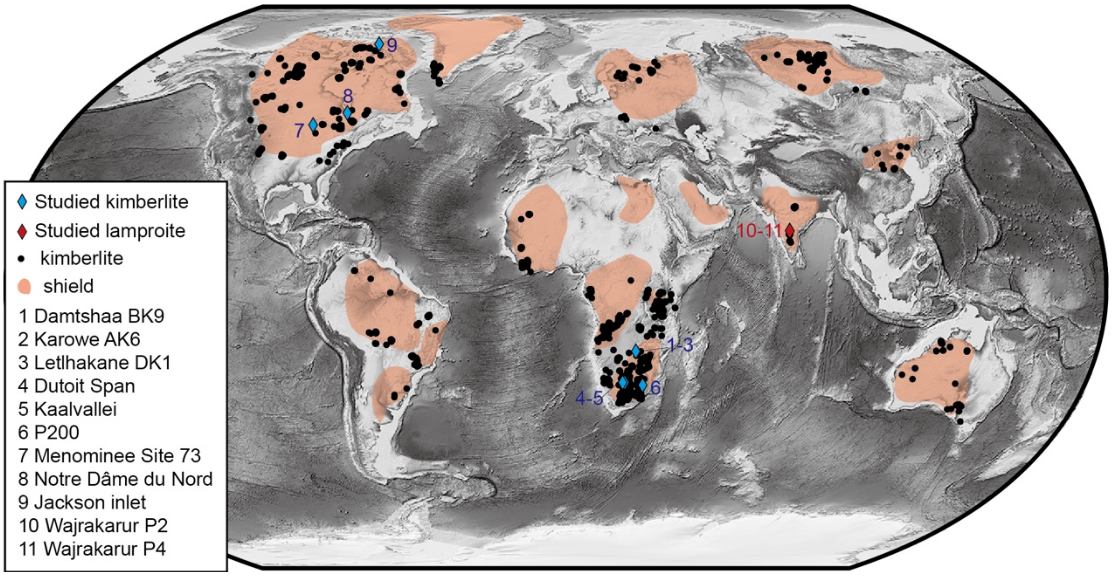

Supplementary Figure 1. Global elevation map showing the distribution of kimberlites, continental shields, and location of studied samples in this work. Modified with the permission of the Mineralogical Society of America from Figure 3 from Guiliani and Pearson (2019) [1], Kimberlites: From Deep Earth to Diamond Mines, Elements, vol 15, 377 - 380.

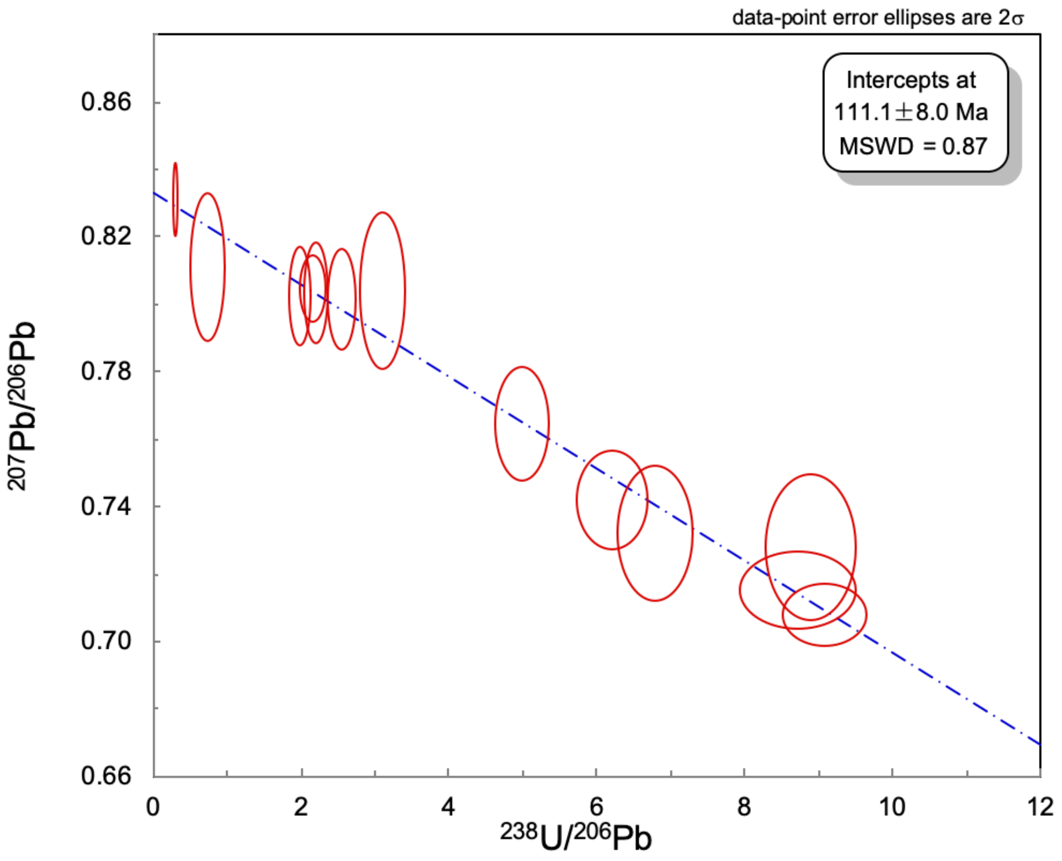

Supplementary Figure 2. U–Pb age concordia diagram for perovskite from the Jackson Inet kimberlite.

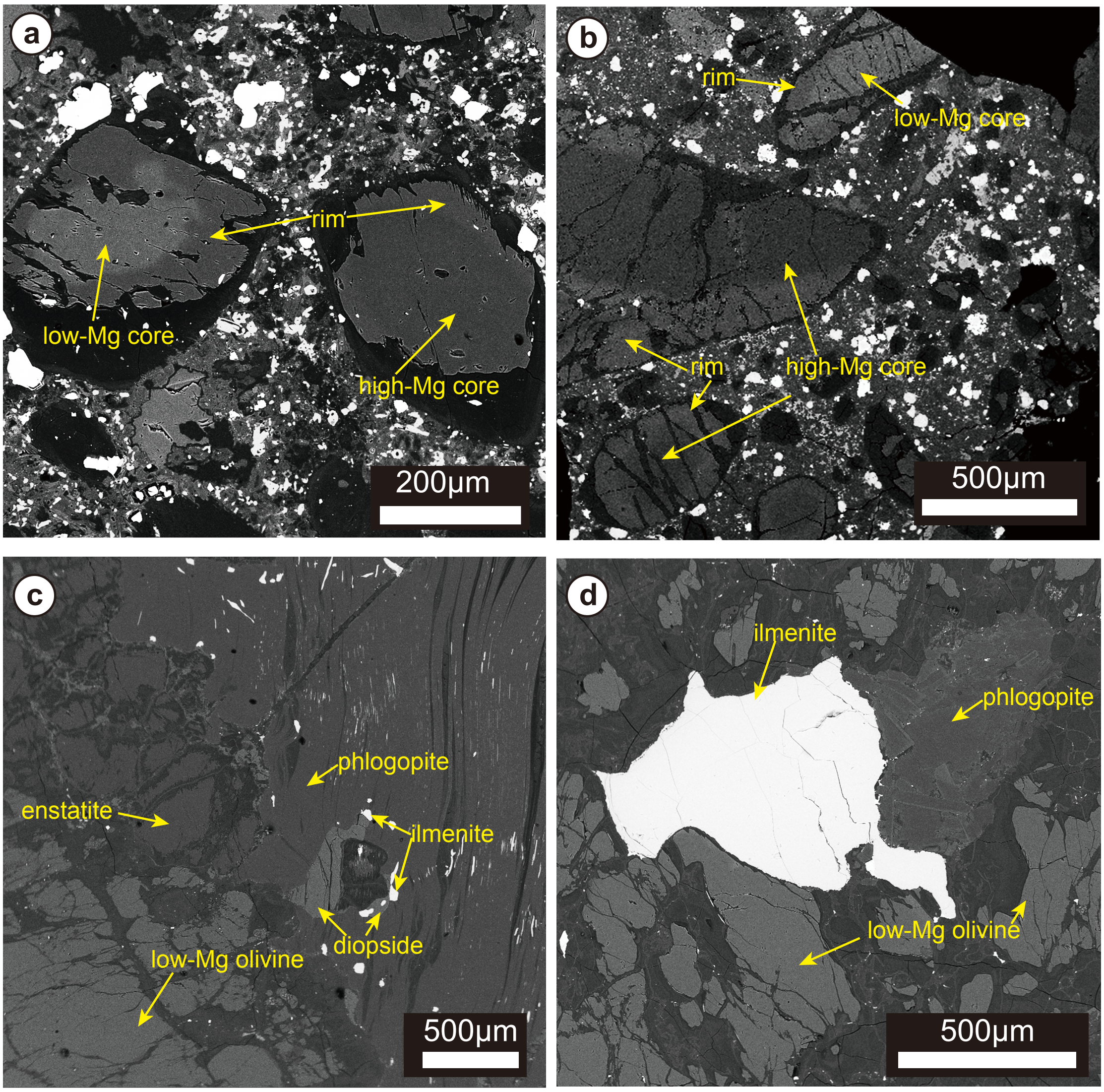

Supplementary Figure 3. Back-scattered electron (BSE) SEM imaging of olivine in kimberlites. **a** Dutoitspan (South Africa); **b** Lethlakane DK1 (Botswana), and (c, d) olivine in PIC-like xenoliths from Damtshaa (Botswana). Note the strong core-rim zoning in kimberlitic olivine.

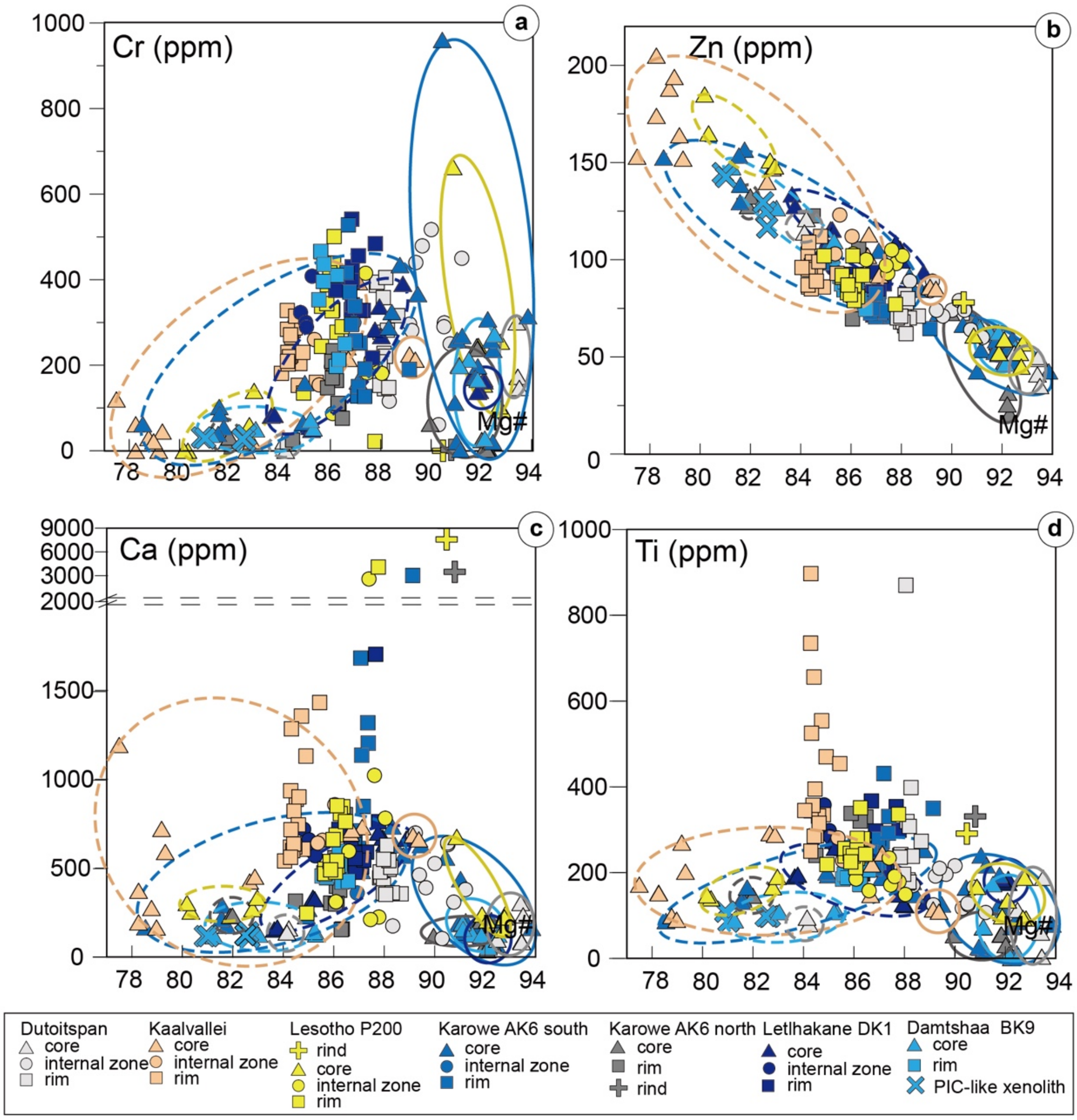

Supplementary Figure 4. Mg# vs (a) Cr, (b) Zn (ppm), (c) Ca, and (d) Ti (ppm) in olivine from the examined southern African kimberlites. Solid lines mark out high-Mg olivine cores, and dashed lines the low-Mg olivine cores.

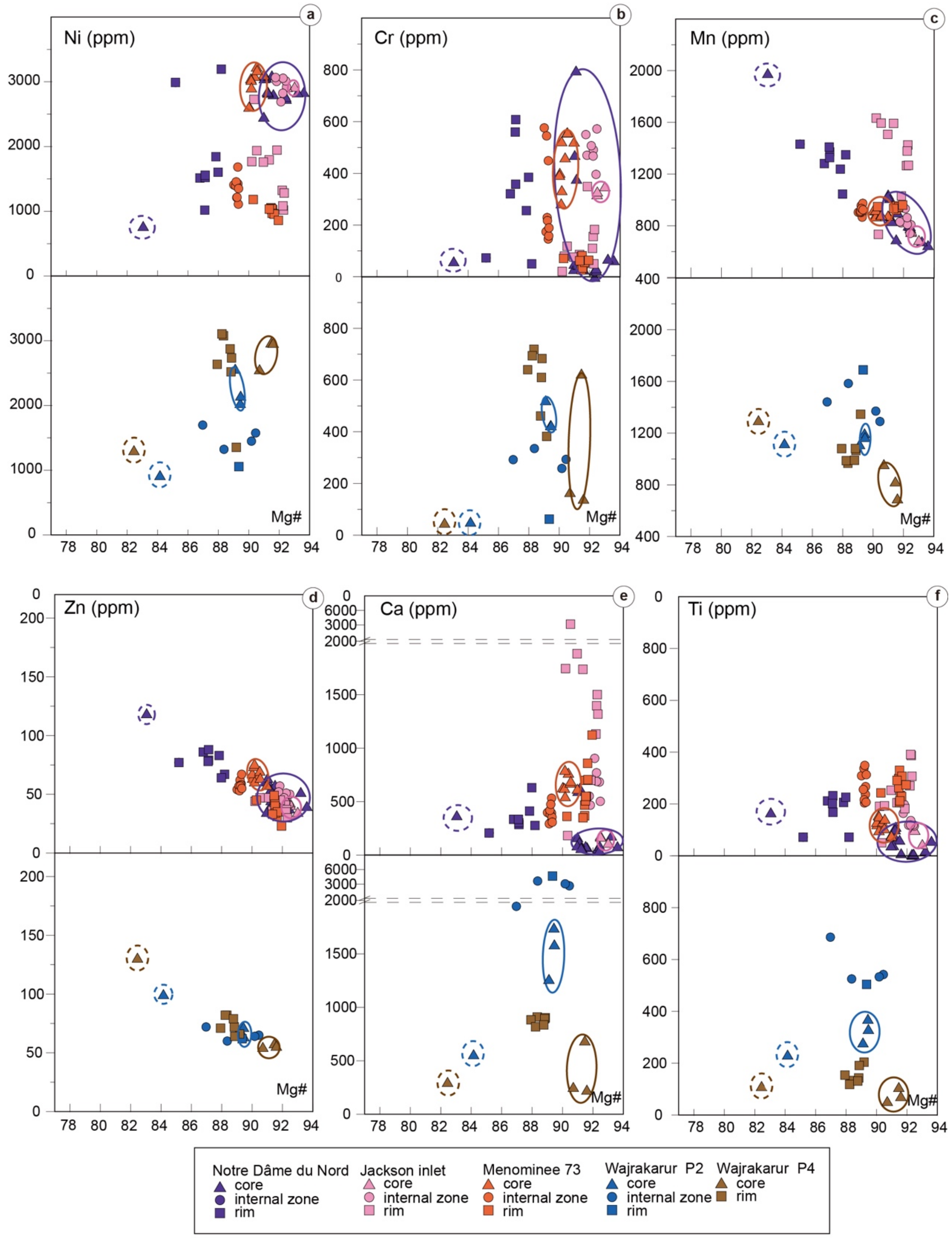

Supplementary Figure 5. Mg# vs (a) Ni, (b) Cr, (c) Mn (ppm), (d) Zn, (e) Ca, and (f) P (ppm) in olivine from the examined kimberlites. Solid lines mark out high-Mg olivine cores, and dashed lines the low-Mg olivine cores.

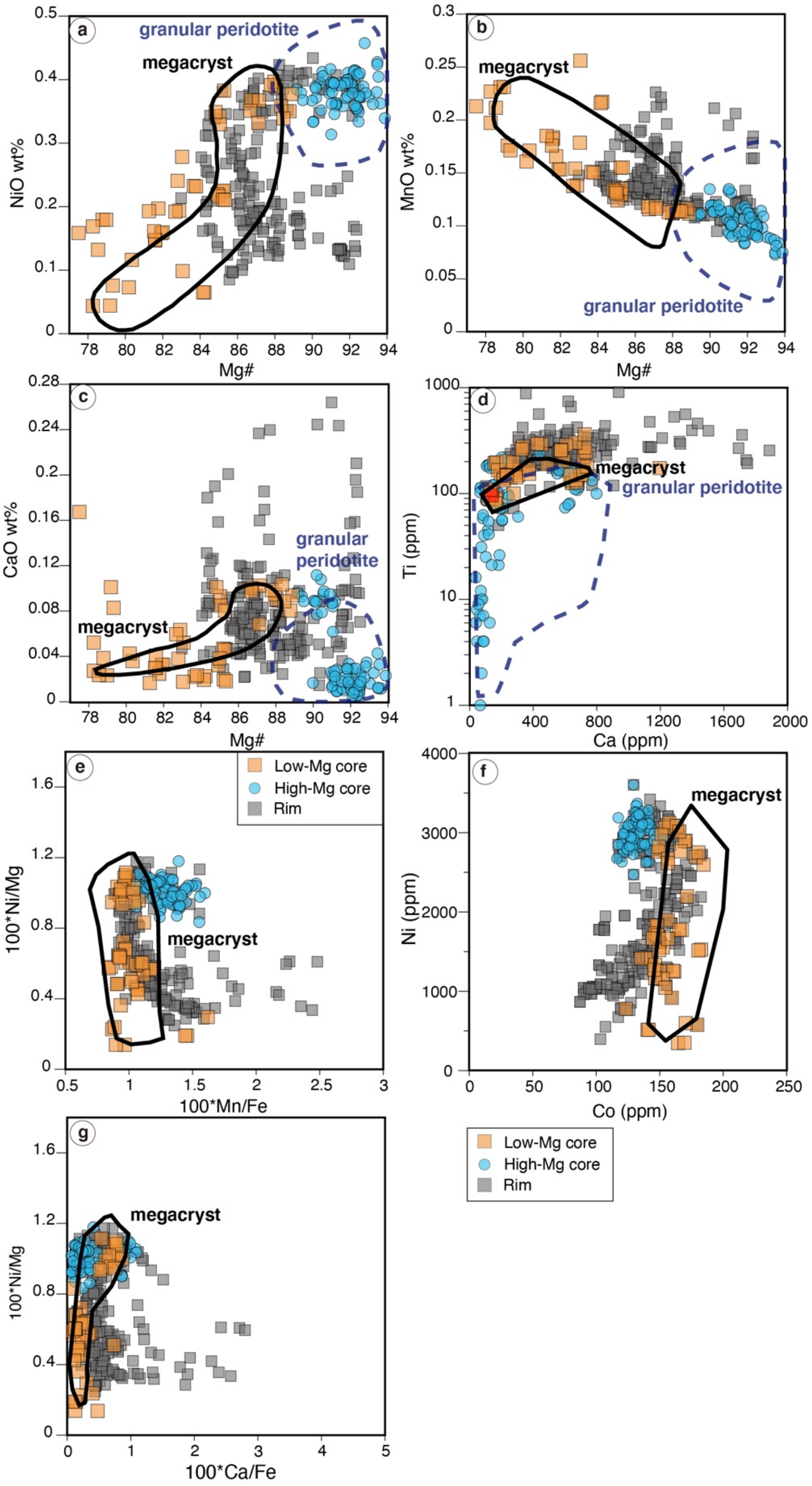

Supplementary Figure 6. Covariation diagrams of (a) Mg# vs. NiO wt.%, (b) Mg# vs. MnO wt.%, (c) Mg# vs. CaO wt.%, (d) Ca vs. Ti (ppm), (e) 100*Mn/Fe vs. 100*Ni/Mg, (f) 100*Ca/Fe vs. 100*Ni/Mg, and (g) Co vs. No (ppm) for olivine cores studied in this work, compared with a field of olivine in granular peridotites and olivine megacrysts [2–4].

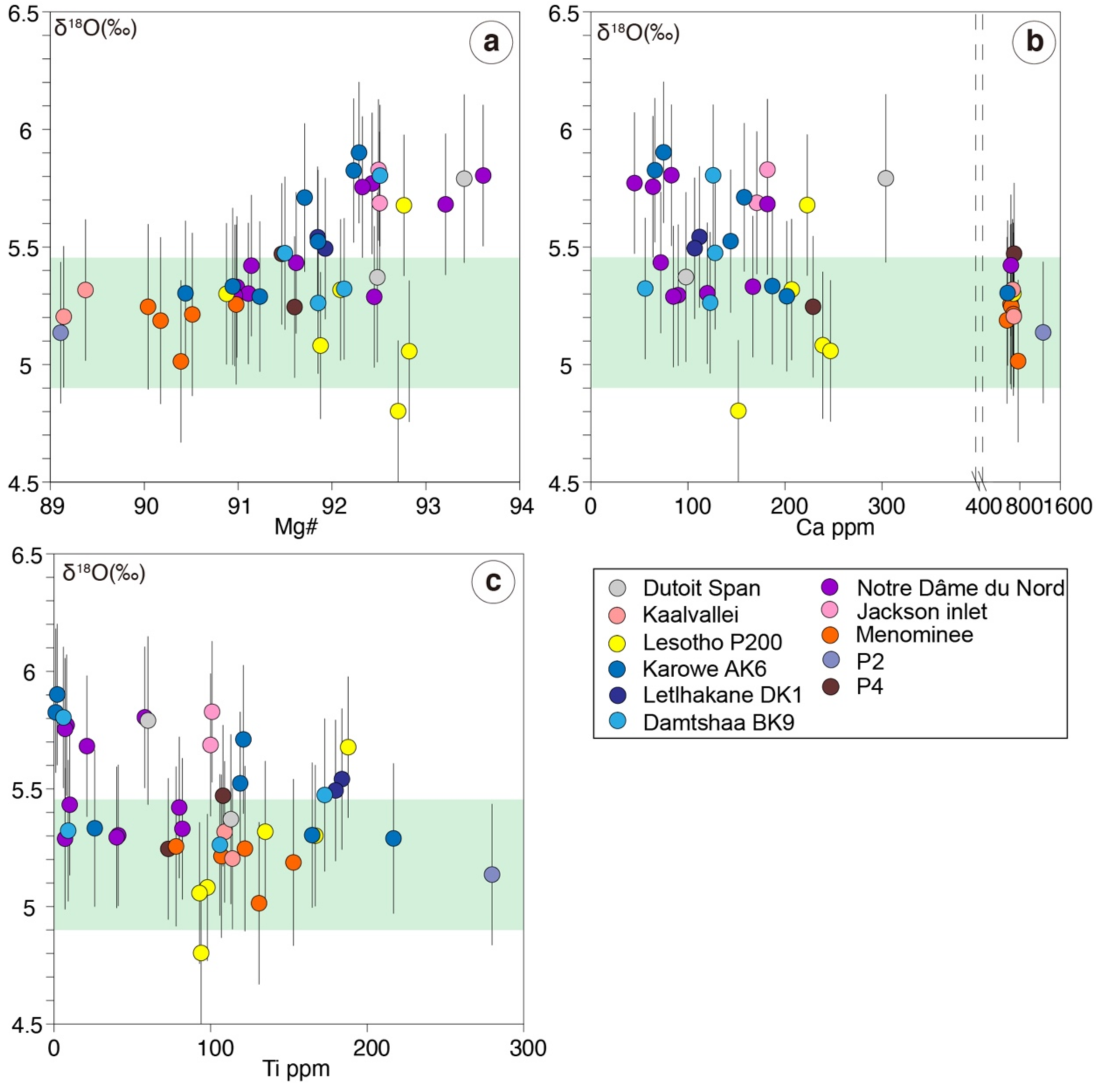

Supplementary Figure 7. Covariation diagram of δ^{18}O versus (a) Mg#, (b) Ca (ppm), (c) Ti (ppm) for high-Mg olivine cores in the examined samples. Green bands represent the mantle olivine value (5.18 ± 0.28‰ [5]). Error bars indicate the 2σ of each analyses.

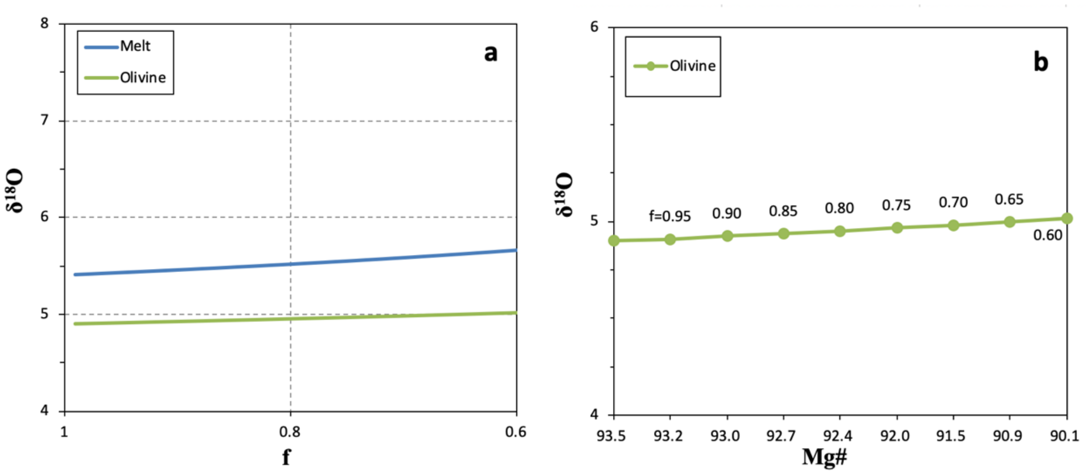

Supplementary Figure 8. Rayleigh fractionation model showing the oxygen isotope evolution of proto-kimberlite melt and crystallized low-Mg (megacrystic) olivine after variable fractionation of olivine, orthopyroxene, clinopyroxene and garnet. See text for model details. (a) Fraction of residual melt (f) vs oxygen isotopes in melt and olivine. (b) Oxygen isotope and Mg# variation of crystallized olivine.

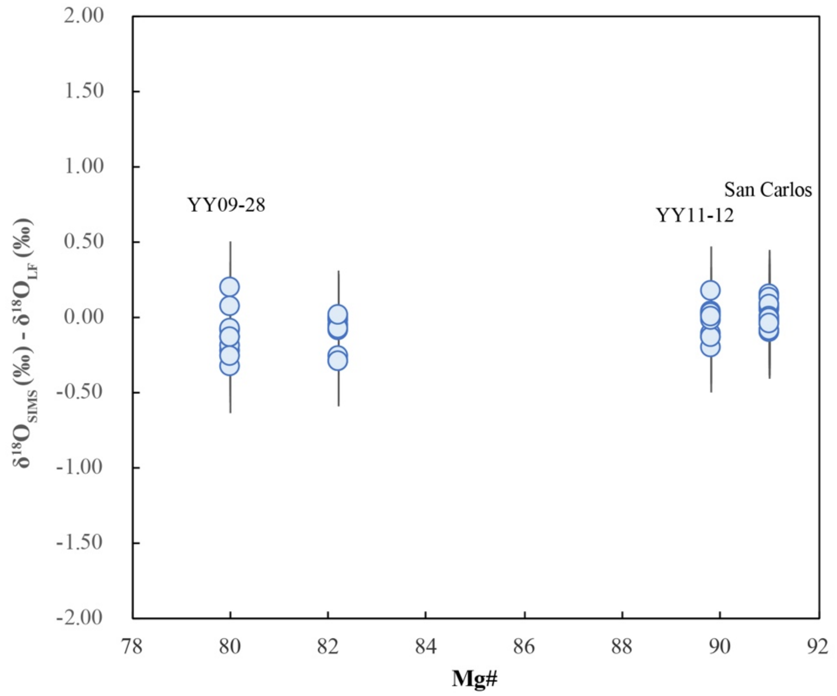

Supplementary Figure 9. Difference between $\delta^{18}O$ analysed by CAMECA IMS 1280 ion probe at IGGCAS using San Carlos olivine as standard and $\delta^{18}O$ analysed by laser fluorination of Yangyuan olivine with different Mg# . Corresponding data is represented in Supplementary material Table 3. Error bars indicate the 2σ of each analyses.

Supplementary References

1. Giuliani, A. & Pearson, D. G. Kimberlites: From Deep Earth to Diamond Mines. *Elements* **15**, 377–380 (2019).
2. Giuliani, A. Insights into kimberlite petrogenesis and mantle metasomatism from a review of the compositional zoning of olivine in kimberlites worldwide. *Lithos* **312–313**, 322–342 (2018).
3. Howarth, G. H. Olivine megacryst chemistry , Monastery kimberlite : Constraints on the mineralogy of the HIMU mantle reservoir in southern Africa. *Lithos* **314–315**, 658–668 (2018).
4. Howarth, G. H. & Taylor, L. A. Multi-stage kimberlite evolution tracked in zoned olivine from the Benfontein sill, South Africa. *Lithos* **262**, 384–397 (2016).
5. Mattey, D., Lowry, D. & Macpherson, C. Oxygen isotope composition of mantle peridotite. *Earth Planet. Sci. Lett.* **128**, 231–241 (1994).
